# Gold Nanoparticles Supported on Ceria Nanoparticles Modulate Leukocyte–Endothelium Cell Interactions and Inflammation in Type 2 Diabetes

**DOI:** 10.3390/antiox11112297

**Published:** 2022-11-20

**Authors:** Pedro Díaz-Pozo, Francisco Canet, Abdessamad Grirrane, Sandra Lopez-Domenech, José Raul Herance, Nadezda Apostolova, Clara Luna-Marco, Susana Rovira-Llopis, Miguel Marti, Carlos Morillas, Milagros Rocha, Hermenegildo Garcia, Victor M. Victor

**Affiliations:** 1 Service of Endocrinology and Nutrition, Foundation for the Promotion of Health and Biomedical Research in the Valencian Region (FISABIO), University Hospital Doctor Peset, 46017 Valencia, Spain; 2 Instituto Universitario de Tecnología Química CSIC-UPV, Universidad Politécnica de Valencia, Avenida de los Naranjos s/n, 46022 Valencia, Spain; 3 Department of Pharmacology, University of Valencia, 46003 Valencia, Spain; 4 Medical Molecular Imaging Research Group, Vall d’Hebron Research Institute, CIBBIM-Nanomedicine, 08035 Barcelona, Spain; 5 Centro de Investigación Biomédica en Red en Bioingeniería, Biomateriales y Nanomedicina CIBERBBN, 28029 Madrid, Spain; 6 Centro de Investigación Biomédica en Red en Enfermedades Hepáticas y Digestivas (CIBEREHD), 46010 Valencia, Spain; 7 Department of Physiology, INCLIVA (Biomedical Research Institute Valencia), University of Valencia, 46010 Valencia, Spain

**Keywords:** gold-ceria nanoparticle, ROS, diabetes, inflammation, atherosclerosis

## Abstract

Gold-ceria nanoparticles (Au/CeO_2_) are known to have antioxidant properties. However, whether these nanoparticles can provide benefits in type 2 diabetes mellitus (T2D) remains unknown. This work aimed to study the effects of Au/CeO_2_ nanoparticles at different rates of gold purity (10, 4.4, 1.79 and 0.82) on leukocyte–endothelium interactions and inflammation in T2D patients. Anthropometric and metabolic parameters, leukocyte–endothelium interactions, ROS production and NF-κB expression were assessed in 57 T2D patients and 51 healthy subjects. T2D patients displayed higher Body Mass Index (BMI) and characteristic alterations in carbohydrate and lipid metabolism. ROS production was increased in leukocytes of T2D patients and decreased by Au/CeO_2_ at 0.82% gold. Interestingly, Au/CeO_2_ 0.82% modulated leukocyte–endothelium interactions (the first step in the atherosclerotic process) by increasing leukocyte rolling velocity and decreasing rolling flux and adhesion in T2D. A static adhesion assay also revealed diminished leukocyte–endothelium interactions by Au/CeO_2_ 0.82% treatment. NF-κB (p65) levels increased in T2D patients and were reduced by Au/CeO_2_ treatment. Cell proliferation, viability, and apoptosis assays demonstrated no toxicity produced by Au/CeO_2_ nanoparticles. These results demonstrate that Au/CeO_2_ nanoparticles at 0.82% exert antioxidant and anti-inflammatory actions in the leukocyte–endothelium interaction of T2D patients, suggesting a protective role against the appearance of atherosclerosis and cardiovascular diseases when this condition exists.

## 1. Introduction

Type 2 diabetes (T2D) is a serious health problem worldwide; the prevalence is increasing and reducing life expectancy [1,2]. It has been demonstrated that T2D is related to cardiovascular risk factors, including obesity, hypertension, dyslipidaemia, insulin resistance and non-alcoholic fatty liver disease [3], though for some, the molecular mechanisms remain to be elucidated.

T2D has been related to oxidative stress and therefore to an increased production of reactive oxygen species (ROS) and, consequently, alterations of the cellular homeostasis and redox state. As the main source of ROS, mitochondria are particularly vulnerable to hyperglycaemic conditions, which lead to enhanced ROS production and oxidative stress [4]. In this sense, oxidative stress and mitochondrial impairment have been related to the onset of insulin resistance, T2D and cardiometabolic diseases [5].

T2D and inflammation are interconnected, playing a key role in host defences; in fact, different studies highlight that the inflammatory process is an important player in the pathogenesis of dyslipidaemia and hyperglycaemia [6]. T2D is a chronic and low-grade inflammatory process that takes place mainly to the presence of high levels of glucose and fatty acids on leukocytes [7,8]. In addition, it has been observed that nuclear factor kappa B (NF-κB), a key player in the inflammatory process, cellular homeostasis and immunity is enhanced under hyperglycaemia [9]. During this process, there is an increased adhesion of leukocytes to the endothelium, after which they migrate in order to eliminate pathogens through the generation of ROS. It is important to mention that an increased ROS production and oxidative stress can promote mitochondrial impairment, which is associated to endothelial dysfunction and eventually in leukocyte–endothelium interactions, inflammatory processes and thrombosis [10]. Therefore, the need for novel therapies that modulate ROS production and oxidative stress in cardiometabolic diseases such as T2D is paramount. In this scenario, the use of nanomaterials to modulate the deleterious effects of T2D offers great potential.

The use of nanoparticles (NPs) in biomedicine is growing, with a variety of potential applications, including antioxidant therapy, bioimaging and drug delivery [11,12,13,14]. Among the different types of NPs, ceria (CeO_2_) NPs are specially promising due to their versatility, biocompatibility and physicochemical properties [15]. Furthermore, it has been demonstrated that CeO_2_ has antioxidant properties and can be used for combating oxidative stress [16,17]. This antioxidant capacity can be increased when NPs are combined with certain metals, including silver, platinum or gold [18,19]. These combinations have demonstrated biocompatible properties by which they act as ROS scavengers and catalase-like enzymes, therefore modulating oxidative stress conditions [20,21,22]. Various human diseases may benefit from nanotherapeutics, and T2D is among them. In biomedical applications, gold NPs (AuNPs) are among the most promising NPs [23]. In this sense, gold nanoparticles have demonstrated beneficial effects in animal models of diabetes by decreasing hepatic enzymes, TNF-α and interleukin-6 [24], or by restoring blood glucose, glycogen and insulin levels [25]. Furthermore, gold nanoparticles can be bioconjugated with metformin, a gold standard treatment for type 2 diabetes, demonstrating beneficial actions by inhibiting the production of advanced glycation end products (AGEs), and by reducing hyperchromicity, early glycation products, carbonyl content, hydxoxymethylfurfural content and production of fluorescent AGEs [26].

Although the aetiology of insulin resistance and T2D is intricate, different studies have reported that ROS can induce oxidative stress and thereby exacerbate insulin resistance [27,28], which highlights the therapeutic potential of antioxidants. In this sense, abundant evidence, both in vitro and in vivo, supports the potential of gold NPs as antioxidants [29] in models of T2D [30], amongst others.

In the present study, we evaluate the potential therapeutic benefits of CeO_2_-supported gold (Au/CeO_2_) NPs with different rates of gold purity in leukocyte–endothelial interactions, and we evaluate their impact on NF-κB in leukocytes from T2D patients.

## 2. Materials and Methods

### 2.1. Patients and Sample Collection

This study is prospective and observational in a cohort of 57 T2D patients—attending the Endocrinology Service of the University Hospital Doctor Peset (Valencia, Spain) and diagnosed according to the American Diabetes Association´s criteria—and 51 matched healthy controls. Patients were recruited according to the standard clinical protocols in the hospital.

The inclusion criteria were aged between 40 and 70 years and an evolution of diabetes of over 10 years. The exclusion criteria were significant renal impairment (creatinine > 1.5 mg/dL or eGFR < 60 mL/min/1.73 m^2^), a smoking habit, frequent alcohol intake, severe diabetic neuropathy, morbid obesity (Body Mass Index; BMI > 40 kg/m^2^) and the presence of chronic diseases other than those directly related to cardiovascular risk. All subjects were informed about the procedures and evaluated parameters and gave their informed written consent. The study was approved from the Hospital’s Ethics Committee (CEIC 98/19) and was performed in compliance with the statement of ethical principles for medical research of the Declaration of Helsinki.

### 2.2. Biochemical Parameters

Venous blood was collected into Vacutainer® tubes in fasting conditions between 8 AM and 10 AM. Samples were then centrifuged for 10 min at 1500× *g* at 4 °C to separate serum from blood. Glucose, total cholesterol, and triglycerides were obtained by an enzymatic method; high-density lipoprotein cholesterol (HDL-c) was determined employing a Beckman LX-20 autoanalyser (Beckman Coulter, La Brea, CA, USA) and low-density lipoprotein cholesterol (LDL-c) was quantified using Friedewald´s formula. Immunochemiluminescence was used to measure insulin levels and HOMA-IR index (fasting insulin (U/mL) fasting glucose (mg/dL)/405). Glycosylated Haemoglobin Type A1C (HbA1c) percentage was determined with a glycohaemoglobin analyser (Arkray Inc., Kyoto, Japan) and high-sensitive C-reactive protein (hs-CRP) levels were evaluated by an immunonephelometric assay.

### 2.3. Leukocyte Isolation

Polymorphonuclear cells (PMN) were obtained from heparinised blood samples. First, PMN were incubated for 45 min with a 1:2 volume of dextran solution (3%) in NaCl (0.9%; Sigma Aldrich, St. Louis, MO, USA), and the supernatant was centrifuged over Fycoll-Hypaque (GE Healthcare, Uppsala, Sweden) for 25 min at 650× *g*. The resulting pellet was then incubated with lysis buffer to eliminate remaining erythrocytes (5 min at RT) and centrifuged at 250× *g*. Finally, pellets containing PMN were washed twice and resuspended with Hank´s balanced salt solution (HBSS; Sigma Aldrich, St. Louis, MO, USA) or Roswell Park Memorial Institute (RPMI) culture medium (Sigma Aldrich, St. Louis, MO, USA), depending on their experimental use. In addition, LUNA-FL (Logos Biosystems Inc., Annandale, VA, USA) was used to determine cell count and viability (acridine orange and propidium iodide double stain). A human macrophage-like (U937) cell line was employed for proliferation and viability studies.

### 2.4. Isolation of Human Endothelial Cells

Human umbilical vein endothelial cells (HUVEC) cells were obtained from the umbilical cords of patients undergoing a normal delivery. Cells were separated using the method previously described by our group [31] and cultured at 5% CO_2_ and 37 °C until they reached pre-confluence. For culture, we used endothelial cell growth medium-2 (EGM-2) containing penicillin, streptomycin and fungizone, which were employed in the culture. HUVEC were then incubated for 3 h with 0.02 mg/mL Au/CeO_2_.

### 2.5. Materials and Instrumentation

Photoluminescence evaluation were conducted in a solution containing acetonitrile at room temperature (RT) in N2-purged septum-capped quartz cells using a Xe-doped mercury lamp, an Edinburgh FL3000 spectrofluorometer (Edinburgh Instruments, Livingston, Scotland) and a Czerny-Turner monochromator (InfoCrops, Shanghai, China).

High-resolution transmission electron microscopy (HR-TEM) images and measurements were performed with JEOL JEM-2100F equipment at the Universidad Politécnica de Valencia, under an accelerating voltage of 200 kV. Samples were prepared by applying one drop of the suspended material in ethanol onto a carbon-coated copper TEM grid and allowing it to dry at RT. X-ray photoelectron spectra (XPS) were measured on a SPECS spectrometer (SPECS Surface Nano Analysis GmbH, Berlin, Germany) equipped with a Phoibos 150-MCD-9 detector and using a non-monochromatic Al K (1486.6 eV) X-ray source. Spectra were recorded at 200 °C, using an analyser pass energy of 30 eV and an X-ray power of 50 W, and the samples were evacuated into the prechamber of the spectrometer at 1 × 10^−9^ mbar. The measured intensity ratios of the components were obtained from the area of the corresponding peaks after nonlinear Shirley-type background subtraction, corrected by the transition function of the spectrometer. During the data processing of the XPS spectra, binding energy (BE) values were referenced to the C1s signal. Spectra treatment was performed using the CASA software (version 6.5, https://casa.nrao.edu/ (accessed on 27 October 2022). Zeta potential and particle size of Au/CeO_2_ particles suspended in neutral H_2_O were measured with a Malvern Zetasizer instrument (Malvern Panalytical Ltd., Malvern, UK) at 5 ppm concentration (see Supplementary File).

Unless otherwise indicated, solvents and reagents were purchased from Sigma-Aldrich and used as received. Synthesis of Au/CeO_2_ was as follows: Preparation of the gold catalysts that were to be supported on the cerium oxide nanoparticulates was carried out by impregnation using the following procedure. A 0.82 wt% Au/CeO_2_ catalyst was prepared by impregnating 5 g of CeO_2_ (Rodhia, Barcelona, Spain) with a solution of 0.90 g of NaAuCl4 in 40 mL of H_2_O (milliQ, Madrid, Spain). The slurry was stirred for 12 h at RT, after which all the liquid was evaporated and the solid dried at 100 °C for 3 h. Next, the solid was reduced and activated by pouring it into boiling 1-phenylethanol (60 mL) at 160 °C for 3 h. The catalyst was then filtered and washed consecutively with water (exhaustive washings with 3 L of distilled water), acetone (100 mL) and diethyl ether (50 mL) and dried under a vacuum at 100 °C for 2 h. Au analysis by XR fluorescence revealed that the Au content was approximately 0.82 wt%, which was close to the optimal value. Other Au/CeO_2_ catalysts containing higher gold concentrations were prepared following the abovementioned procedure but using larger quantities of NaAuCl_4_.

Four batches of nanoparticles were prepared with varying amounts of gold (10, 4.4, 1.79 and 0.82), expressed as a% of the total weight at the Institute of Chemical Technology (ITQ, Valencia, Spain).

### 2.6. Evaluation of Leukocyte–Endothelium Interactions

First, we evaluated the anti-inflammatory capacity of NPs with different amounts of Au (10, 4.4, 1.79 and 0.82), expressed as a % of the total weight (Au/CeO_2_) at the same concentration (0.02 mg/mL). We employed 1.2 mL of T2D and control peripheral blood PMN with a 10^6^ cell/mL density in complete RPMI. Three/four days prior to the experiment, primary cultures of HUVEC were prepared. PMN were passed along the HUVEC monolayer at a velocity of 0.3 mL/min during 5 min and were recorded throughout. Therefore, the leukocyte rolling velocity was evaluated by measuring the time taken by the PMN to cover 200 micrometres. PMN rolling flux was evaluated over a 1 min period and adhesion was determined by measuring the PMN adhering to the endothelium in 5 separate fields over a period of at least 30 s.

### 2.7. Au/CeO_2_ Catalyst Preparation

Preparation of the catalysts on the CeO_2_ nanoparticles by impregnation was performed following the procedure, exemplified by a particular Au loading: 0.82, 1.79, 4.4 and 10 wt%. Au/CeO_2_ catalysts were prepared by impregnating 5 g of CeO_2_ with a solution of 0.90 g NaAuCl_4_ in 40 mL ultrapure H_2_O. The slurry was stirred for 12 h at RT, after which all the liquid was allowed to evaporate. The remaining solid was dried at 100 °C for 3 h, and was then reduced and activated by pouring it into boiling 1-phenylethanol (at 160 °C), where it was kept for 3 h. The catalyst was then filtered and washed consecutively by water (exhaustive washings with distilled water: 3 L), acetone (100 mL) and diethyl ether (50 mL) and dried under a vacuum at 100 °C for 2 h. Au analysis by quantitative fluorescence X-ray emission revealed that the Au contents were close to the expected value according to the NaAuCl_4_ amount added to the solution. Dynamic laser scattering of the Au/CeO_2_ NPs dispersed in neutral aqueous solutions revealed a mononodal particle size distribution of an average size of 4.562 and 6.1856 nm for the 0.82 and 1.79 wt%. Au/CeO_2_ catalysts, respectively (See the Supplementary Material). The electrostatic potentials for agglomeration were −31.9, −17.1 mV for 0.82 and 1.79 Au loading, respectively, indicating that these suspensions are persistent in water, particularly for the lowest Au loading.

### 2.8. Static Leukocyte–Endothelial Interaction Assay

We performed a modified version of the leukocyte–endothelial monolayer adhesion assay [32]. First, PMN were isolated and labelled with 2 µM 2′, 7′-Bis-(2-carboxyethyl)-5 (and-6) carboxyfluorescein, acetoxymethyl ester (BCECF-AM) (Thermo Fisher Scientific, Waltham, MA, USA) to a final concentration of 2 × 10^6^ cells/mL. Simultaneously, HUVEC were seeded in 96-well microtiter tissue-culture plates (Corning, Glendale, United States)) and cultured until they reached pre-confluence. A total of 3 h prior to the assay, HUVEC were treated with DMSO (0.141 M), TNF-α (5 ng/mL) and Au/CeO_2_ NPs (0.02 mg/mL). HUVEC were then washed once with RPMI + 1% Fetal bovine serum (FBS). Next, 100 µL of the BCECF-AM labelled PMN suspension was added to each well and allowed to interact with HUVEC for 45 min. After this, PMN were removed, and each well was washed three times with HBSS to eliminate any non-adhering PMN. Finally, the well contents were solubilised in 150 µL of lysis buffer (50 mM Tris-HCl, 0.1% SDS in water, pH 8.2–8.4) and the plates were measured by the microplate reader Synergy Mx (BioTek Instruments GmbH, Bad Friedrichshall, Germany).

### 2.9. Cellular Viability and Proliferation

U937 (ATCC® CRL1593.2 ™) cells were seeded in 24-well plates at 100.000 cells/well in RPMI supplemented with 10% FBS. Treatment with Au/CeO_2_ or DMSO was administered over 3 h, after which the medium was refreshed, and the U937 proliferated for 3 days. At 24, 48 and 72 h, the cells were analysed with LUNA-FL to assess cell number and viability (acridine orange and propidium iodide double stain). All treatments were performed in triplicate.

### 2.10. Evaluation of Apoptosis

U937 cells were seeded in 24-well plates at 500,000 cells/well in RPMI supplemented with 10% FBS. Treatment with Au/CeO_2_ or DMSO was administered over 3 h, after which the medium was refreshed, and cells were cultured for a further 21 h. The well-known apoptosis inducer staurosporine (STS) was used as a positive control for cell death. The FITC Annexin V Apoptosis Detection Kit I (BD Biosciences, CA, USA) was employed for the assay. Cells were washed twice with cold phosphate-buffered saline (PBS) and resuspended in 1× binding buffer at a concentration of 1 × 10^6^ cells/mL. Subsequently, 100 µL of the solution (1 × 10^5^ cells) were transferred to a 5 mL culture tube, to which 5 µL of FITC Annexin V and 5 µL of propidium iodide were added. The presence of four populations were evaluated: apoptotic (AnnV+/PI−), vital (AnnV−/PI−), late apoptotic/necrotic (AnnV+/PI+), and damaged cells (AnnV−/PI+).

### 2.11. Measurement of Total ROS Production

PMN were seeded in 48-well plates and were treated after 24 h with the NPs from the cell culture media at a concentration of 0.02 mg/mL for 3 h. Total ROS production was evaluated by fluorescence (IX81 Olympus, Hamburg, Germany) after 30 min incubation in HBSS (GIBCO, Thermo Fisher Scientific, Sant Cugat del Vallés, Spain) with 5 µM Hoechst 33342 (for nuclei) and 5 µM 2’7’–dichlorodihydrofluorescein diacetate (DCFH–DA for ROS) (both from Sigma-Aldrich, Madrid, Spain). Therefore, cells were washed twice with HBSS and a total of 20–30 images per well were recorded with a fluorescence microscope coupled with static cytometry software (“ScanR” version 2.03.2, IX81 Olympus Europa, Hamburg, Germany). Controls were performed by using HBSS.

### 2.12. Western Blotting

First, PMN were isolated as described in Section 2.3 and resuspended to a final concentration of 1 × 10^6^ cells/mL. Simultaneously, HUVEC were seeded in 25 cm 2 flask and cultured until they reached pre-confluence. 3 h prior to the assay, HUVEC were treated with 0.82% Au/CeO_2_ nanoparticles (0.02 mg/mL). Next, 5 mL of the PMN suspension was added to each flask and allowed to interact with HUVEC for 3 h. After this time, PMN and HUVEC were stored separately. Afterwards, HUVEC pellets were incubated in RIPA Buffer during 15 min (Thermo Fisher Scientific, Waltham, MA, USA) with a protease inhibitor cocktail (Merck Life Science S.L.U., Madrid, Spain). Tubes were then vortexed and sonicated 3 times, for 10 s each time, to disrupt cell membranes, and were centrifuged at 14,000× *g* for 15 min. Supernatants were stored at −80 °C until further use. A BCA protein assay kit (Thermo Fisher Scientific, Sant Cugat del Vallés, Spain) was used for protein concentration determination and 25 µg protein per sample were loaded onto 4–20% SDS-polyacrylamide gels. Gel electrophoresis was performed at 150 V, for 90 min, after which proteins were transferred to nitrocellulose membranes (Bio-Rad, Hercules, CA, USA) at 400 mA for 1 h. Membranes were then blocked at RT for 1 h in 5% non-fat milk in TBS-T buffer containing 150 mM sodium chloride, 0.1% Tween20 and 25 mM Tris at pH 7.5. After eliminating the excess blocking buffer, membranes were incubated overnight at 4 °C with anti NF-κB rabbit polyclonal antibody (Abcam, Cambridge, UK) and anti-actin mouse polyclonal antibody (Cell Signaling, Danvers, MA, USA), and subsequently with horseradish peroxidase goat anti-rabbit (Millipore Iberica, Madrid, Spain) or horseradish peroxidase (HRP) goat anti-mouse (Thermo Fisher Scientific, Sant Cugat del Vallés, Spain) secondary antibodies, as appropriate, for 1 h at RT. After removing the excess of antibody, protein expression was evaluated with a ECL plus reagent (GE Healthcare, Amersham Place, Litte Chalfont, UK) or Supersignal West Femto (Thermo Fisher Scientific, Sant Cugat del Vallés, Spain) using the Fusion FX5 acquisition system (Vilbert Lourmat, Marne La Vallée, France) for chemiluminescence signal detection. Data were analysed by densitometry using Bio1D software (Vilbert Lourmat, Marne La Vallée, France). All protein expression values were normalised to those of actin in the same sample and an internal control was included in each blot.

### 2.13. Statistical Analysis

Data analysis was performed with SPSS 25.0 (SPSS Statistics Inc., Chicago, IL, USA). Results for parametric and non-parametric variables were expressed as mean ± SD and median (25th–75th percentiles), respectively. Statistical analysis was performed with a Student’s *t*-test for normally distributed data, Mann–Whitney U test for non-normally distributed values, and a chi-square test for proportion of frequencies. In addition, the influence of BMI on biochemical parameters was evaluated with an univarate general linear model using BMI as a covariate. Differences were considered statistically significant when *p* < 0.05. Graphs were plotted with GraphPad Prism 6.0 (GraphPad, La Jolla, CA, USA).

## 3. Results and Discussion

### 3.1. Clinical and Endocrine Characteristics of the Study Subjects

A total of 51 T2D patients were analysed and compared with 57 age- and sex-matched healthy subjects. Biochemical and anthropometric parameters are shown in Table 1.

T2D patients displayed an altered carbohydrate metabolism with respect to the control group. Glucose, insulin, HOMA-IR and HbA1c levels were significantly higher than among controls (*p* < 0.001). In addition, higher values of upper waist circumference (*p* < 0.001), BMI, weight and hs-CRP were observed in the T2D group (*p* < 0.001). In terms of lipid profile, a lower HDL-c level (*p* < 0.001) and a higher triglyceride concentration (*p* < 0.001) were characteristics of our T2D patients; however, due to the presence of lipid-lowering therapy involving statins (64%), total cholesterol (*p* < 0.001) and LDL-c levels (*p* < 0.001) were slightly lower. When statistical significance was recalculated after adjusting for BMI, all the parameters’ differences remained statistically significant, except for triglycerides and hs-CRP.

### 3.2. Characterisation of Nanoparticles

**Figure 1:**
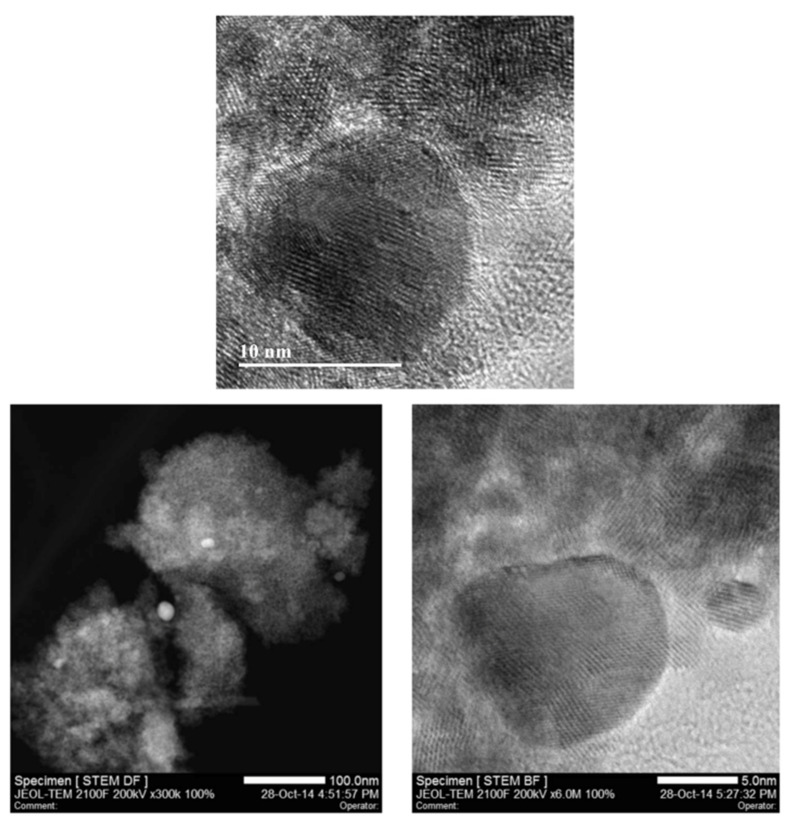
Representative HR-TEM (**Top**), dark Field (**Bottom**, **Left**) and bright Field (**Bottom**, **Right**) HR-STEM images of the 0.82% Au/CeO_2_ catalyst. The high-resolution image allows one to distinguish the crystal planes corresponding to Au particles. Au element is well distributed in the particles, as observed in the bright field image.

We have characterised the NPs with the following techniques. Figure 1 shows the selected HR-TEM and STEM images for a sample of 0.82 wt% Au/CeO_2_ with the presence of Au NPs.

**Figure 2:**
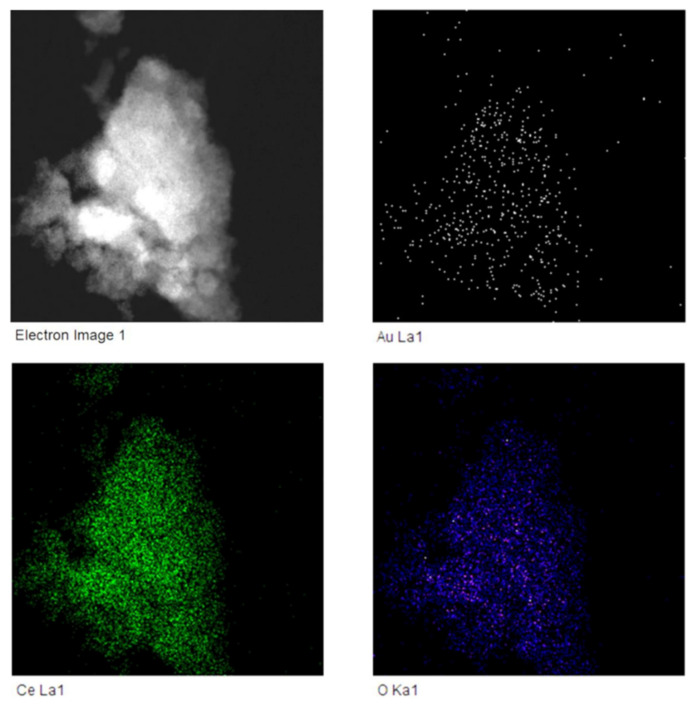
Dark field scanning electron microscopy of 0.82% AuCeO_2_ catalyst and EDX elemental mapping of gold, cerium and oxygen demonstrating that these elements are evenly distributed in the particle.

Therefore, we performed a dark field scanning electron microscopy of 0.82% AuCeO_2_ catalyst and EDX elemental mapping. Elemental mapping at the microscopic level of this catalyst (presented in Figure 2) revealed a homogeneous dispersion of Au on the CeO_2_ support in a sample of 0.82 wt% Au/CeO_2_; i.e., there was a similar distribution of both elements.

**Figure 3:**
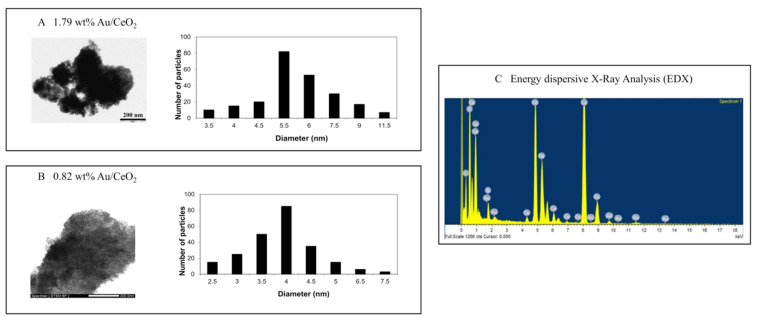
HR-STEM images and corresponding histograms of nanoparticle size distribution for (**A**) 1.79% AuCeO_2_ with average diameter 6.04 nm and (**B**) 0.82 wt% Au/CeO_2_ (**B**) with average diameter 3.94 nm. (**C**) Energy dispersive X-Ray analysis (EDX) demonstrating the presence in the particles of Au and Ce.

In addition, we evaluated HR-STEM images and corresponding histograms of nanoparticle size distribution (Figure 3).

**Figure 4:**
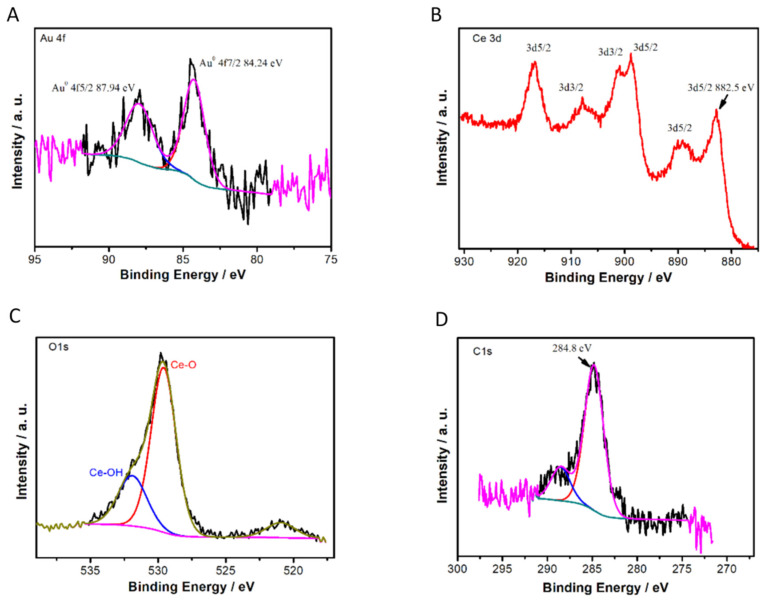
Experimental high resolution XPS peaks, best deconvolutions and assignments of Au 4f (**A**), Ce 3d (**B**), O1s (**C**) regions for a sample of 0.82% Au/CeO_2_ and C1s (**D**) XPS spectra used as reference.

Finally, in Figure 4: (A) Gold peaks of the XPS spectrum corresponding to the Au 4f region in a sample of 0.82 wt% Au/CeO_2_ and the corresponding deconvolution, indicating the contribution of Au0 with a binding energy of 84.24 and 87.94 eV; (B) the Ce 3d XPS region showing the contribution mainly of Ce4+; (C) O1s XPS spectra with two different bands, at 529.41 and 531.91 eV, associated to CeO and CeOH, respectively; and (D) C1S XPS spectra, used as a charge reference having a typical binding energy of 284.8 eV corresponding to the C-C component.

### 3.3. Leukocyte–Endothelium Interactions

**Figure 5:**
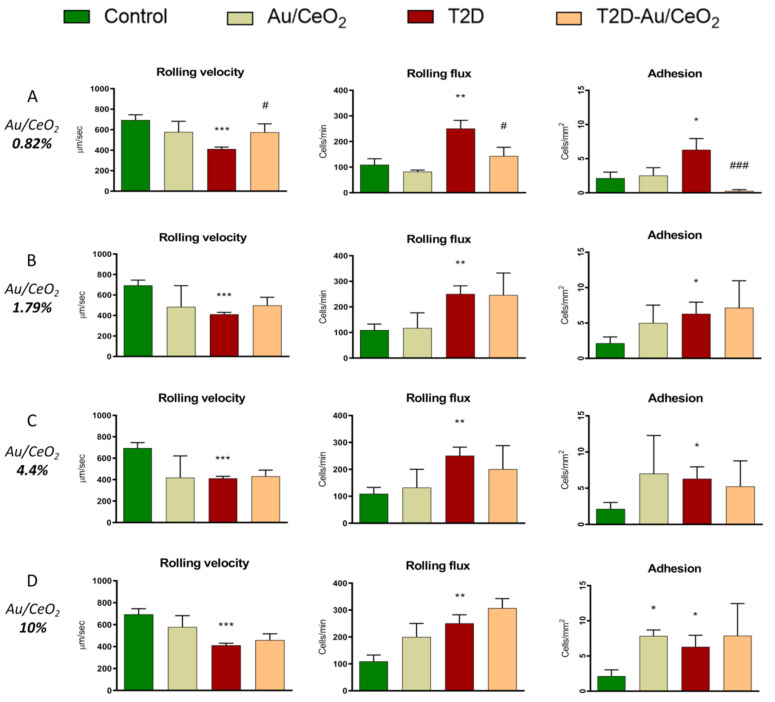
Evaluation of leukocyte–endothelium interaction in presence and absence of Au/CeO_2_ (3 h, 0.02 mg/mL) in T2D patients and control subjects. Rolling velocity (μm second^−1^), rolling flux (leukocytes per minute) and adhesion (leukocytes per square millimetre). * *p* < 0.05 vs. control ** *p* < 0.01 vs. control; *** *p* < 0.001 vs. control. # *p* < 0.05 vs DM2; ### *p* < 0.001 vs DM2.

It is known that levels of many proinflammatory markers are increased in patients with metabolic disorders. Therefore, we assessed the influence of T2D and Au/CeO_2_ on the interaction of PMN with the endothelial monolayer, one of their main functions. In fact, the interaction between leukocytes to the endothelium can be the first step in the atherosclerotic process. We observed a reduction in leukocyte rolling velocity (*p* < 0.001, Figure 5A–D), and increased leukocyte adhesion (*p* < 0.05, Figure 5A–D) and rolling flux (*p* < 0.01, Figure 5A–D) in diabetic patients with respect to control individuals. Treatment with ceria NPs containing 10, 4.4 and 1.79 wt% Au did not alter these effects (Figure 5B–D). Importantly, treatment with 0.82 wt% Au/CeO_2_ reversed the effect observed in leukocyte rolling velocity (*p* < 0.05, Figure 5A), rolling flux (*p* < 0.05, Figure 5A) and leukocyte adhesion (*p* < 0.05, Figure 5A). In agreement with these results, it has been demonstrated that the administration of CeO_2_ nanoparticles can protect the heart from oxidative and inflammatory injury induced by the cardiac-specific expression of monocyte chemotactic protein-1 (MCP-1) [33]. In line with these ideas, the possible beneficial effects for combating atherosclerosis with targeted nanomedicines has been reviewed [34].

Given that the effect of these particles on the leukocyte–endothelium interaction was only observed with 0.82 wt% Au, and that NPs with a higher proportion of Au (1.79, 4.4 or 10%) lacked an effect, we carried out the remaining experiments with 0.82 wt% Au. It is known that Au loading determines the activity of Au/CeO_2_ as a catalyst. On the one hand, higher Au loadings should increase the number of sites. On the other hand, high loadings increase the Au average particle size, decreasing the catalytic activity. Due to these opposite effects, an optimal Au loading in the range from 0.1 to 2 wt% is frequently observed. In the present case, it seems that the performance of Au (0.82 wt%)/CeO_2_ is better than the Au (1.79 wt%)/CeO_2_ at higher Au loading. We can also speculate that particles with different amounts of Au penetrate the cellular membranes to a varying extent, thus modulating their biological activity. In fact, it is important to highlight the importance of size-dependent cytoprotective effects of nanoparticles, as it has been recently described [35]. In this study, they demonstrate that selenium nanoparticles (SeNPs) with a diameter of 100 nm have a more pronounced cytoprotective effect on the cells of the cerebral cortex under conditions of ischaemia/reoxygenation (OGD/R) through an increased baseline and an OGD/R-induced expression of genes encoding protective proteins, suppression of the OGD-induced global increase in [Ca^2+^]i and activation of reactive astrogliosis. Whereas 50 nm-sized and 400 nm-sized SeNPs, while also exerting a protective effect, do not eliminate the OGD-induced hyperexcitation of neurons. They conclude that one of the explanations for this difference in the effectiveness of the cytoprotective action of different-sized SeNPs is the presence of two endocytosis pathways for large SeNPs (100 and 400 nm) and the presence of some toxic effects in 50 nm-sized SeNPs.

**Figure 6:**
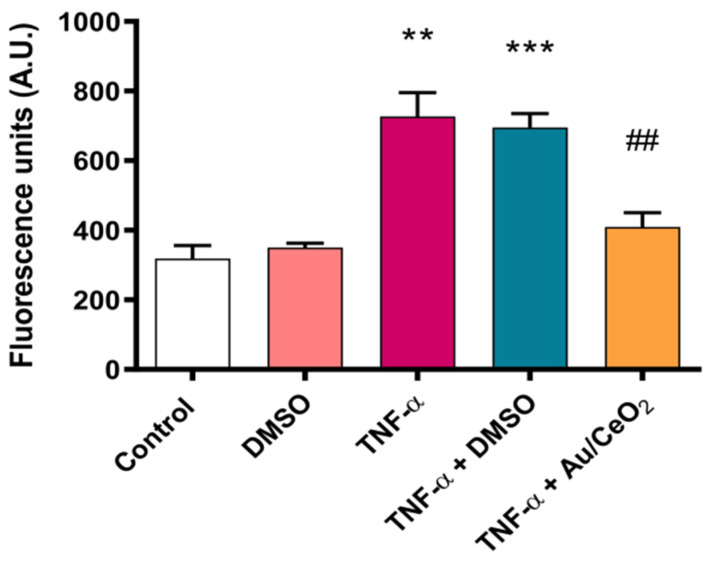
Au/CeO_2_ (3 h, 0.02 mg/mL) effects in leukocyte adhesion to HUVEC monolayer stimulated with TNF-α (5 ng/mL). ** *p* < 0.01 vs. not stimulated; *** *p* < 0.001 vs. not stimulated; ## *p* < 0.01 vs. stimulated.

To further explore interactions between leukocytes and endothelial cells, we performed a static interaction assay (Figure 6). Data showed a significant increase in leukocyte adhesion when HUVEC were stimulated with TNF-α (5 ng/mL) (*p* < 0.01). DMSO did not alter leukocyte adhesion in unstimulated or stimulated HUVEC. Interestingly, treatment with 0.82 wt% Au/CeO_2_ nanoparticles (0.02 mg/mL for 3 h) reversed TNF-α-induced adhesion (Figure 6), resulting in values similar to those of unstimulated cells.

### 3.4. Biocompatibility of Au/CeO_2_

**Figure 7:**
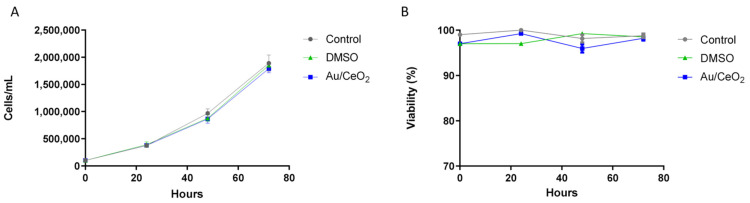
Determination of cellular proliferation and viability. Effect of Au/CeO_2_ and its vehicle DMSO on U937 cell line. (**A**) Cell count over 3 days revealed no difference in cellular proliferation between each condition over the course of the experiment (72 h). (**B**) Cell viability assessed with acridine orange and propidium iodide double stain.

When exploring the effects of an antioxidant agent, it is crucial to assess its biocompatibility. This is particularly important in the case of Au/CeO_2_, which is a catalyst with strong oxidation activity. However, it should be reminded that the remarkable catalytic activity is observed in the presence of oxygen at atmospheric or higher pressure and frequently upon heating. Since the reaction rate is first order with respect to oxygen pressure, this catalytic activity should be very attenuated under the present conditions. For the biocompatibility study, we used the U937 cell line (human monocytes) to evaluate the following parameters: (i) cell viability, (ii) proliferation and (iii) induction of apoptosis. Regarding the viability and proliferation assay, cells treated with DMSO or 0.82 wt% Au/CeO_2_ demonstrated a similar multiplication rate to that of untreated cells, maintaining the same percentages of proliferation (Figure 7A). Viability was not affected by any of the treatments (Figure 7B).

**Figure 8:**
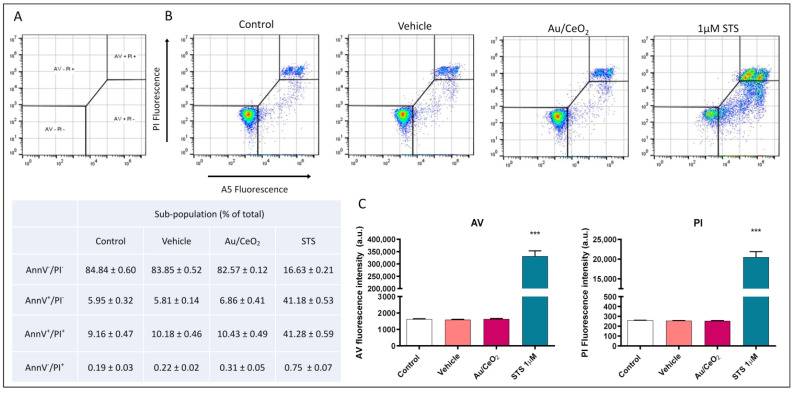
Assessment of apoptosis in U937 cells. (**A**) Diagram of the gating showing all the populations for each histogram. (**B**) Representative histograms (Bivariate Annexin V/PI analysis) of untreated cells, vehicle (DMSO), Au/CeO_2_ treatment and positive apoptotic control (1 μM STS) demonstrating the existence of 4 cellular sub-populations: AnnV−/PI−, AnnV+/PI−, AnnV−/PI+ and AnnV+/PI+. Table shows the percentage of each sub-population for every condition studied (Mean + SEM). (**C**) Summary of Annexin V and PI fluorescence data. Data (mean + SEM) were analysed by Student´s *t* test. Significance *** *p* < 0.001 vs. Untreated.

These results were confirmed by specifically studying apoptosis performing an Annexin V and propidium iodide bivariate assay (Figure 8). We evaluated four different cellular subpopulations: Vital (AnnV−/PI−), apoptotic (AnnV+/PI−), late apoptotic (AnnV−/PI+) and necrotic (AnnV+/PI+). Nearly 85% of the STS treated cells displayed apoptotic features: compared to 15% of negative control (untreated cells). The portion of apoptotic cells in the vehicle and Au/CeO_2_ were similar (16.2% and 17.4%, respectively) (Figure 8A,B) and demonstrated no difference when compared to untreated cells. These results revealed no increase in the proportion of apoptotic or necrotic cells due to the treatment of any of the particles analysed (Figure 8C).

Therefore, our cell toxicity analysis has revealed that 0.82 wt% Au NPs do not compromise cell viability. This is in line with previously published data demonstrating that Au-supported CeO_2_ NPs possess a high degree of biocompatibility with RAW 264.7 macrophage cells [36]. Furthermore, and in agreement with these results, the lack of apoptosis induction in HeLa cells incubated with the NPs by studying nuclear morphology has been demonstrated, i.e., chromatin condensation and nuclear fragmentation, which are two typical apoptotic features [22].

### 3.5. ROS Production and Levels of NF-κB (p65)

Multiple targets could be proposed to mediate the antioxidant and anti-inflammatory effects of gold, ceria and Au-coated ceria NPs on the leukocyte–endothelium system. CeO_2_ NPs have been reported to reduce the production of ROS and suppress iNOS protein levels in stimulated macrophages [37], and to diminish mRNA levels of several inflammatory markers including IL-8, intercellular adhesion molecule 1 (ICAM-1) and monocyte chemotactic protein 1 (MCP-1) in human aortic endothelial cells (HAECs) [38].

**Figure 9:**
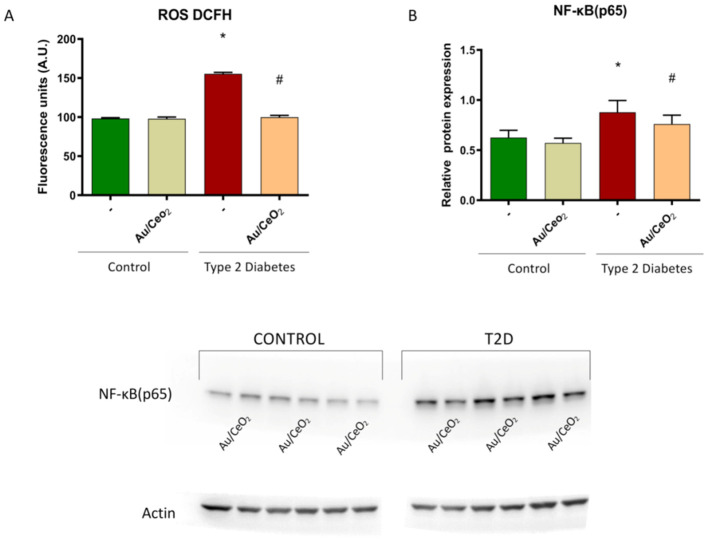
(**A**) ROS production evaluated by DCFH-DA, (**B**) levels of NF-κB (p65) treated or not with Au/CeO_2_ (3 h, 0.02 mg/mL) in leukocytes from type 2 diabetic patients and control subjects and (**C**) representative WB image. * *p* < 0.05 vs. control group. # *p* < 0.05 vs. T2D group (original WB are shown in Supplementary Figure S1).

ROS can play a key role in hyperglycaemia-mediated endothelial impairment and vascular complications associated to insulin resistance and T2D [39,40]. In fact, it has been demonstrated that T2D patients have an enhanced risk of developing cardiovascular events, which is probably related to mitochondrial ROS production and oxidative stress [31]. We examined ROS levels by means of fluorescence and observed that leukocytes from T2D patients displayed a higher amount of ROS compared to those from control subjects (*p* < 0.05) (Figure 9A). In addition, the treatment with Au/CeO_2_ containing 0.82 wt% Au significantly reduced ROS levels (*p* < 0.05) (Figure 9A), pointing to a beneficial antioxidant effect in T2D. In line with our findings, the antioxidant capacity of Au/CeO_2_ has been previously described in HeLa cells [41].

T2D is related to inflammation and, therefore, to proinflammatory cytokines [42]. In fact, it has been demonstrated that T2D increases NF-κB in leukocytes, highlighting the importance of this transcription factor [43]. In this sense, we examined the early cellular immune response by analysing the protein expression of NF-κB (subunit p65). HUVEC incubated with PMN from T2D patients displayed an increased amount of p65 compared to those incubated with PMN from control subjects (*p* < 0.05) (Figure 9B,C). Moreover, treatment with Au/CeO2 containing 0.82 wt% Au significantly reduced the rise in NF-κB (p65) expression (*p* < 0.05) (Figure 9B,C), suggesting a protective anti-inflammatory effect of Au/CeO_2_ NP. In agreement with these results, it has been described that AuNPs can ameliorate muscle atrophy by reducing hyperglycaemia, inflammation and oxidative stress, and by suppressing the ubiquitin-proteasome proteolytic process in an animal model of diabetes [44]. In this sense, it has been demonstrated that other antioxidant compounds, such as MitoQ [45] or MitoTempoL, can protect leukocytes and pancreatic β-cells against oxidative stress by decreasing NF-κB activity and promoting their survival in T2D [46].

## 4. Conclusions

In the present study, we demonstrate a beneficial role of Au/CeO_2_ 0.82% nanoparticles in T2D, especially by decreasing leukocytes/endothelium interactions. In general, we suggest that oxidative stress and increased inflammation, together with NF-κB activation, contribute to the enhanced interactions between these cells, which in turn increase the risk of cardiovascular diseases. Importantly, we demonstrate that Au/CeO_2_ 0.82% are biocompatible, and modulate these actions, thus decreasing ROS production and NF-κB, which endorses the beneficial effects of this compound in preventing cardiovascular diseases in T2D.

## Figures and Tables

**Table 1:** Anthropometric and biochemical parameters.

	Control	T2D	*p*-Value	BMI Adjusted *p*-Value
*n*	57	51		
Age (years)	53.7 ± 7.7	57.10 ± 10.3	NS	
Weight (kg)	72.8 ± 18.0	84.7 ± 16.4	<0.001	
BMI (kg/m^2^)	25.7 ± 4.14	30.6 ± 5.48	<0.001	
Waist (cm)	87.7 ± 12.7	103.5± 11.7	<0.001	
Glucose (mg/dL)	94.1 ± 9.4	153.7± 45.0 ***	<0.001	<0.001
Insulin (μL/mL)	7.19± 2.62	25.9± 15.7 ***	<0.001	<0.001
HOMA-IR	1.67 ± 0.70	9.74 ± 6.87 ***	<0.001	<0.001
HbA_1c_ (%)	5.31 ± 0.34	7.14 ± 1.16 ***	<0.001	<0.001
TC (mg/dL)	203.7 ± 34.5	158.7 ± 39.1 ***	<0.001	<0.001
HDL-c (mg/dL)	57.2 ± 19.7	43.5 ± 9.85 **	<0.001	<0.003
LDL-c (mg/dL)	126.5 ± 29.5	87.0 ± 31.7 ***	<0.001	<0.001
TG (mg/dL)	90.5 (62.8–150.5)	132 (92.8–164.75) **	<0.009	NS
Hs-CRP (mg/L)	1.17 (0.44–2.17)	2.45 (1.22–5.39)	<0.001	NS

Values are expressed as mean ± standard deviation for parametric data and as median (25th and 75th percentiles) for non-parametric data. The three groups were compared with one-way analysis of variance (and Student–Newman–Keuls post hoc test) or the Kruskal–Wallis test (and Dunn’s multiple-comparison post hoc test) for parametric and non-parametric data, respectively. The analysis of covariance was performed with a univariate general linear model using the BMI as a covariate. ** *p* < 0.01 and *** *p* < 0.001 with respect to control. Abbreviations: BMI, body–mass index; HbA1c, glycated haemoglobin A1c; HDL-c, high-density lipoprotein cholesterol; HOMA-IR, homeostasis model assessment of insulin resistance; hs-CRP, highsensitive C-reactive protein; LDL-c, low-density lipoprotein cholesterol; T2D, type 2 diabetes; TC, total cholesterol; TG, triglycerides; NS: not significant.

## Data Availability

The data are contained within this article and Supplementary Files.

## Supplementary Materials

The following supporting information can be downloaded at https://www.mdpi.com/article/10.3390/antiox11112297/s1, Supplementary File includes information about Zeta potential, Figure S1: Original WB pictures.
