# Supplementary material for: Gold Nanoparticles Supported on Ceria Nanoparticles Modulate Leukocyte–Endothelium Cell Interactions and Inflammation in Type 2 Diabetes
Source: Antioxidants (Basel). 2022 Nov 20;11(11):2297. doi: 10.3390/antiox11112297 (PMC9686981; doi:10.3390/antiox11112297)

Size Distribution Report by Volume

v2.0

Malvern Instruments Ltd - © Copyright 2008

Sample Details

Sample Name: 1.79% Au/CeO2_0.7 mg 1

SOP Name: mansettings.nano

General Notes:

**File Name:**	1.79% Au/CeO2_0.7 mg.dts	**Dispersant Name:**	Water
**Record Number:**	1	**Dispersant RI:**	1,330
**Material RI:**	1,36	**Viscosity (cP):**	0,8872
**Material Absorbtion:**	0,01	**Measurement Date and Time:**	jueves, 27 de octubre de ...

System

**Temperature (°C):**	25,0	**Duration Used (s):**	70
**Count Rate (kcps):**	170,3	**Measurement Position (mm):**	4,65
**Cell Description:**	Disposable sizing cuvette	**Attenuator:**	8

Results

			Diam. (nm)	% Volume	Width (nm)
**Z-Average (d.nm):**	487,9	**Peak 1:**	410,7	0,1	203,3
**PdI:**	0,578	**Peak 2:**	4678	1,9	1015
**Intercept:**	0,179	**Peak 3:**	4,562	98,0	1,385

Result quality **Refer to quality report**

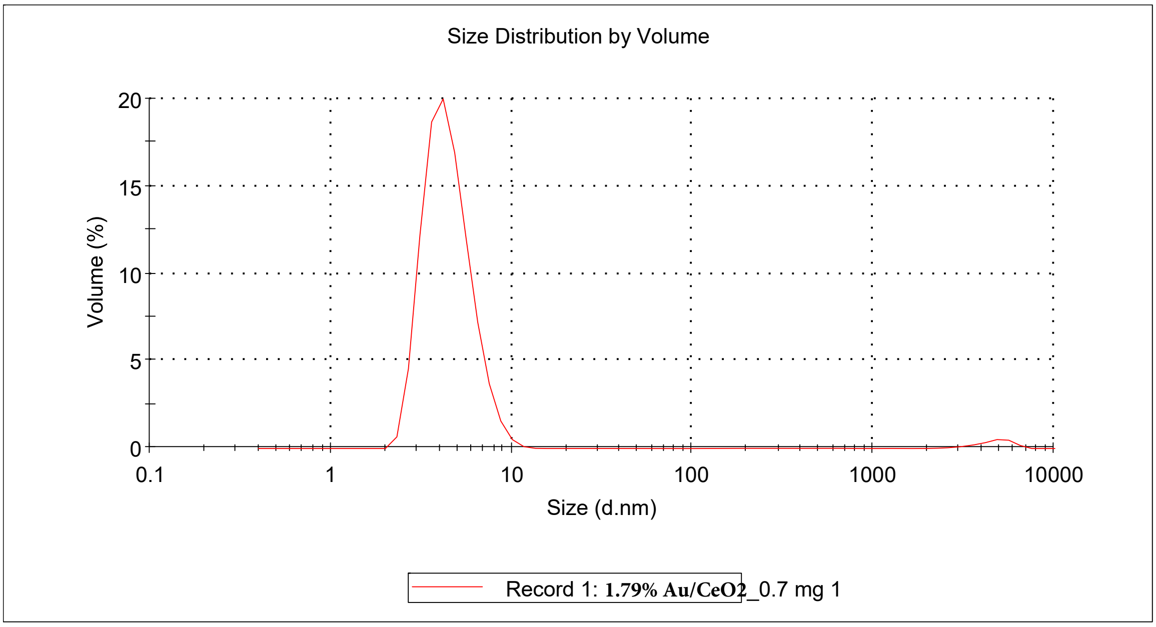

Zeta Potential Report

v2.2

Malvern Instruments Ltd - © Copyright 2008

Sample Details

**Sample Name:**	1.79%AuCeO2-H2O_AVG
**SOP Name:**	1.79%AuCeO2-H2O.sop
**General Notes:**	Average result created from record number(s): 5 12 13

**File Name:**	1.79% Au/CeO2_0.7 mg.dts	**Dispersant Name:**	Water
**Record Number:**	16	**Dispersant RI:**	1,330
**Date and Time:**	jueves, 27 de octubre de 2022...	**Viscosity (cP):**	0,8872
		**Dispersant Dielectric Constant:**	78,5

System

**Temperature (°C):**	25,0	**Zeta Runs:**	15
**Count Rate (kcps):**	87,6	**Measurement Position (mm):**	2,00
**Cell Description:**	Clear disposable zeta cell	**Attenuator:**	11

Results

			Mean (mV)	Area (%)	Width (mV)
**Zeta Potential (mV):**	-17,1	**Peak 1:**	-15,7	100,0	6,50
**Zeta Deviation (mV):**	5,70	**Peak 2:**	0,00	0,0	0,00
**Conductivity (mS/cm):**	0,00350	**Peak 3:**	0,00	0,0	0,00

Result quality Good

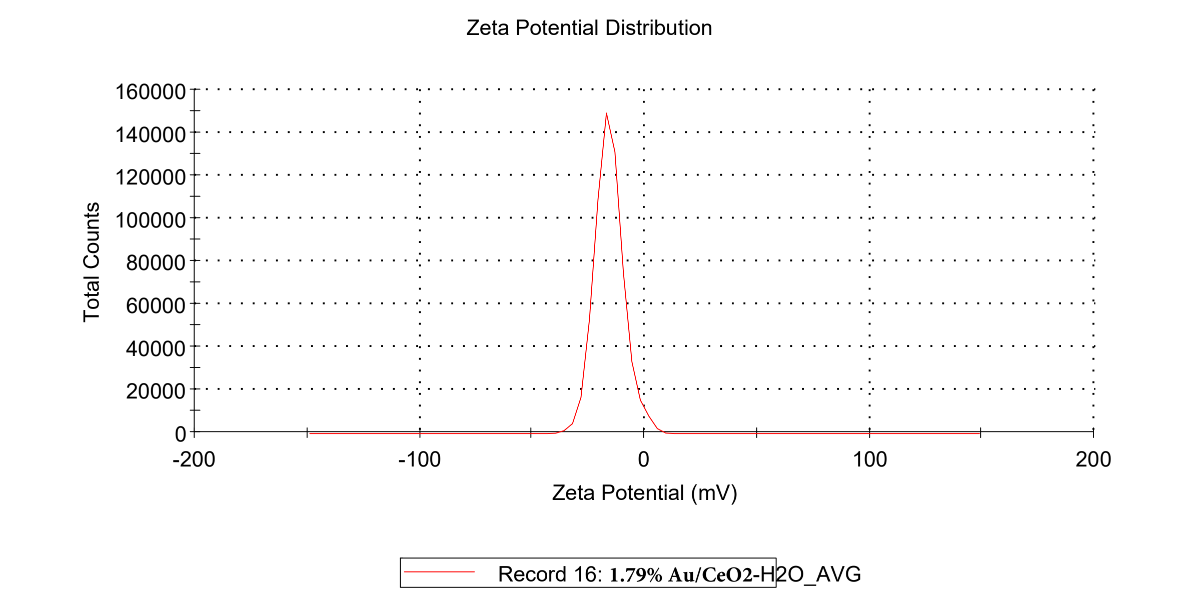

Zeta Potential Report

v2.2

Malvern Instruments Ltd - © Copyright 2008

Sample Details

Sample Name: 0.82% Au/CeO2_0.7 mg_AVG

SOP Name: 0.82% Au/CeO2-H2O.sop

General Notes: Average result created from record number(s): 9 11 13

**File Name:**	0.82% Au/CeO2_0.7 mg.dts	**Dispersant Name:**	Water
**Record Number:**	21	**Dispersant RI:**	1,330
**Date and Time:**	jueves, 27 de octubre de 2022...	**Viscosity (cP):**	0,8872
		**Dispersant Dielectric Constant:**	78,5

System

**Temperature (°C):**	25,0	**Zeta Runs:**	14
**Count Rate (kcps):**	15,0	**Measurement Position (mm):**	2,00
**Cell Description:**	Clear disposable zeta cell	**Attenuator:**	11

Results

			Mean (mV)	Area (%)	Width (mV)
**Zeta Potential (mV):**	-31,9	**Peak 1:**	-31,1	100,0	5,66
**Zeta Deviation (mV):**	5,99	**Peak 2:**	0,00	0,0	0,00
**Conductivity (mS/cm):**	0,00213	**Peak 3:**	0,00	0,0	0,00

Result quality Good

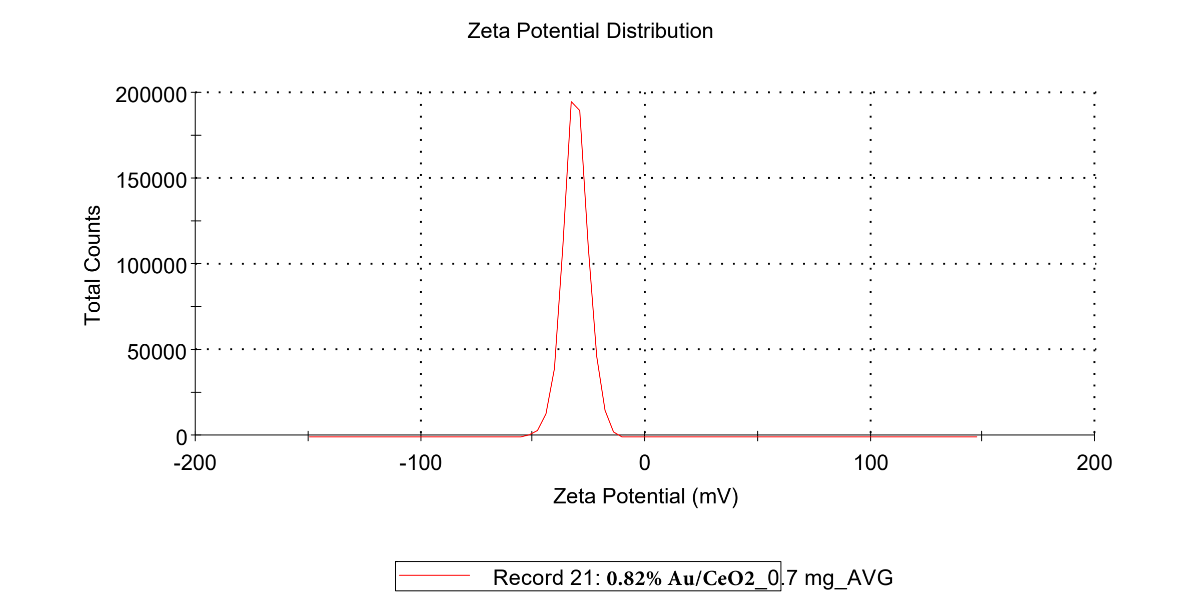

Size Distribution Report by Volume

v2.0

Malvern Instruments Ltd - © Copyright 2008

Sample Details

**Sample Name:**	0.82% Au/CeO2_0.7 mg 3
**SOP Name:**	0.82% Au/CeO2-H2O.sop
**General Notes:**	

**File Name:**	0.82% Au/CeO2_0.7 mg.dts	**Dispersant Name:**	Water
**Record Number:**	3	**Dispersant RI:**	1,330
**Material RI:**	1,83	**Viscosity (cP):**	0,8872
**Material Absorbtion:**	0,01	**Measurement Date and Time:**	jueves, 27 de octubre de ...

System

**Temperature (°C):**	25,0	**Duration Used (s):**	70
**Count Rate (kcps):**	33,7	**Measurement Position (mm):**	4,65
**Cell Description:**	Disposable sizing cuvette	**Attenuator:**	10

Results

			Diam. (nm)	% Volume	Width (nm)
**Z-Average (d.nm):**	239,5	**Peak 1:**	308,3	0,2	168,3
**PdI:**	0,420	**Peak 2:**	5109	0,2	818,0
**Intercept:**	0,428	**Peak 3:**	6,185	99,6	1,395
**Result quality**	**Refer to quality report**				

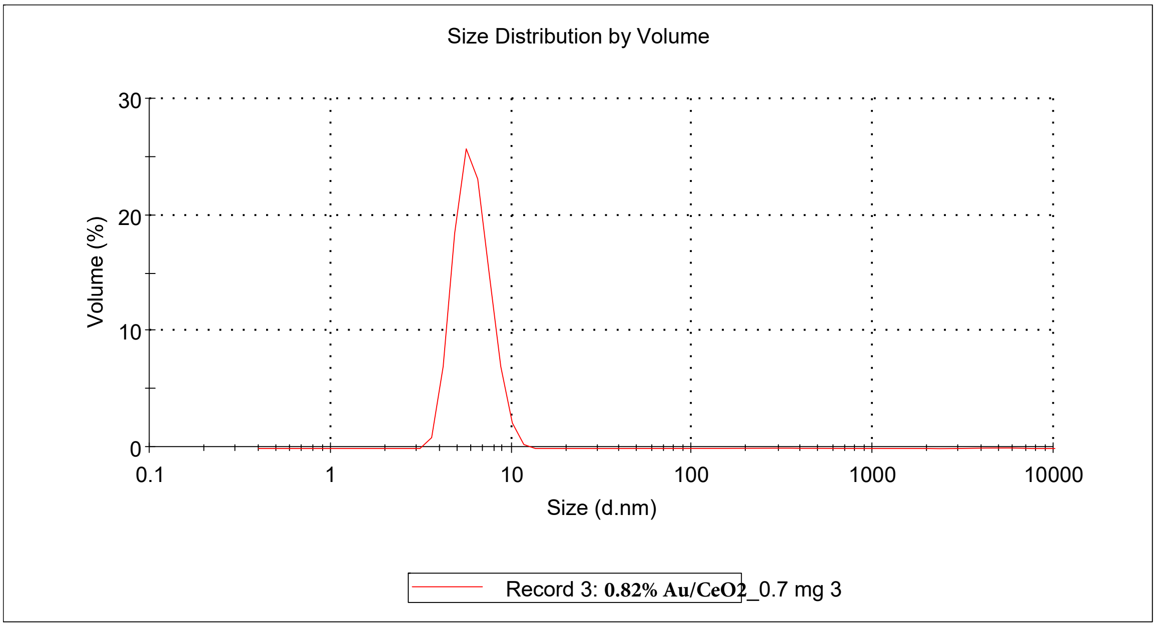

Supplementary Figure S1

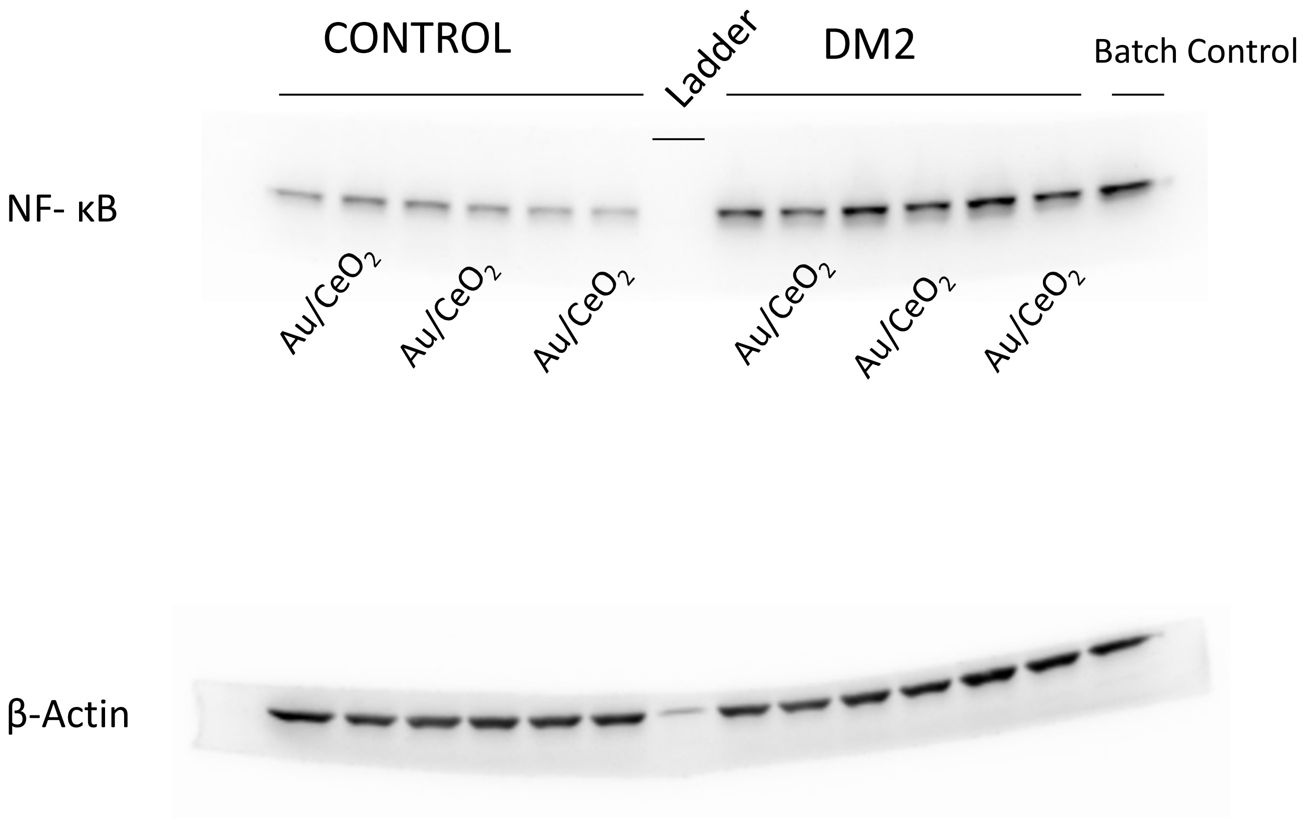

Supplement: Supplementary file 1 [file antioxidants-11-02297-s001.zip › antioxidants-1986484-supplementary.pdf]
